# End-to-End Deep Convolutional Recurrent Models for Noise Robust Waveform Speech Enhancement

**DOI:** 10.3390/s22207782

**Published:** 2022-10-13

**Authors:** Rizwan Ullah, Lunchakorn Wuttisittikulkij, Sushank Chaudhary, Amir Parnianifard, Shashi Shah, Muhammad Ibrar, Fazal-E Wahab

**Affiliations:** 1 Wireless Communication Ecosystem Research Unit, Department of Electrical Engineering, Chulalongkorn University, Bangkok 10330, Thailand; 2 Department of Physics, Islamia College Peshawar, Peshawar 25000, Pakistan; 3 National Engineering Laboratory for Speech and Language Information Processing, University of Science and Technology of China, Hefei 230026, China

**Keywords:** E2E speech processing, Convolutional Encode-Decoder, Convolutional Recurrent Network, speech quality, intelligibility

## Abstract

Because of their simple design structure, end-to-end deep learning (E2E-DL) models have gained a lot of attention for speech enhancement. A number of DL models have achieved excellent results in eliminating the background noise and enhancing the quality as well as the intelligibility of noisy speech. Designing resource-efficient and compact models during real-time processing is still a key challenge. In order to enhance the accomplishment of E2E models, the sequential and local characteristics of speech signal should be efficiently taken into consideration while modeling. In this paper, we present resource-efficient and compact neural models for end-to-end noise-robust waveform-based speech enhancement. Combining the Convolutional Encode-Decoder (CED) and Recurrent Neural Networks (RNNs) in the Convolutional Recurrent Network (CRN) framework, we have aimed at different speech enhancement systems. Different noise types and speakers are used to train and test the proposed models. With LibriSpeech and the DEMAND dataset, the experiments show that the proposed models lead to improved quality and intelligibility with fewer trainable parameters, notably reduced model complexity, and inference time than existing recurrent and convolutional models. The quality and intelligibility are improved by 31.61% and 17.18% over the noisy speech. We further performed cross corpus analysis to demonstrate the generalization of the proposed E2E SE models across different speech datasets.

## 1. Introduction

Applications that are connected to speech signals, such as Automated Speech Recognition (ASR), voice signal communication, speaker verification, and hearing aids, all play a significant part in contemporary societies. The speech along with noise signals are captured by the sound sensors (microphones) where speech enhancement enables the above-mentioned applications to work effectively in noisy environments. Nevertheless, the vast majority of these apps are not resilient when dealing with interference. As a result, speech enhancement (SE) [1,2,3,4], a technique that tries to enhance the intelligibility and quality of the original speech signals, has seen widespread use in the context of these applications. Over the last few years, deep learning techniques have seen an increased amount of use when it comes to the construction of SE systems. Enhancement of the frequency-domain acoustic properties is carried out by a subset of SE systems, which fall under the category of what are known as spectral-mapping-based SE method types. In these methods [5,6,7,8], short-time Fourier transform (STFT) and inverse short-time Fourier transform (inverse STFT) are used to analyse and reconstruct speech signals, respectively. Then, the deep learning models, namely fully connected deep denoising auto-encoder [9], convolutional neural networks (CNNs) [10], recurrent neural networks (RNNs) [11] and long short-term memory (LSTM) [12,13], are utilized as a transformation function to change the noise degraded spectral features to clean features. Moreover, in the meantime, various techniques are being developed by integrating various kinds of deep learning models (for example, CNN and RNN) in order to more efficiently obtain the local and sequential correlations [14,15].

In recent past, an SE system that was developed based on stacked simple recurrent units (SRUs) [16,17] has demonstrated denoising performance comparable to that of the LSTM-based SE system while needing significantly fewer computational costs for training. This was accomplished by an SE system that was based on stacked simple recurrent units (SRUs) [16]. Due to the absence of proper phase information, the augmented speech signal will never be able to realize its full potential, despite the fact that the methodologies described above are currently capable of providing remarkable performance. Some SE systems use complex-spectral-mapping and complex-ratio-masking to improve distorted speech [18,19]. This is conducted in order to combat the issue that was just described. In [20], the phase estimation was recast as a classification issue, and it was used in the process of source separation. A further category of SE techniques offers the opportunity to directly conduct augmentation on the raw waveform in [21,22]. These methods, which are typically referred to as waveform-mapping-based approaches, are classified as a subcategory of SE methods. Fully convolutional networks, often known as FCNs, are one kind of deep learning model that has seen widespread use for the purpose of directly performing waveform mapping [23,24].

**Figure 1:**
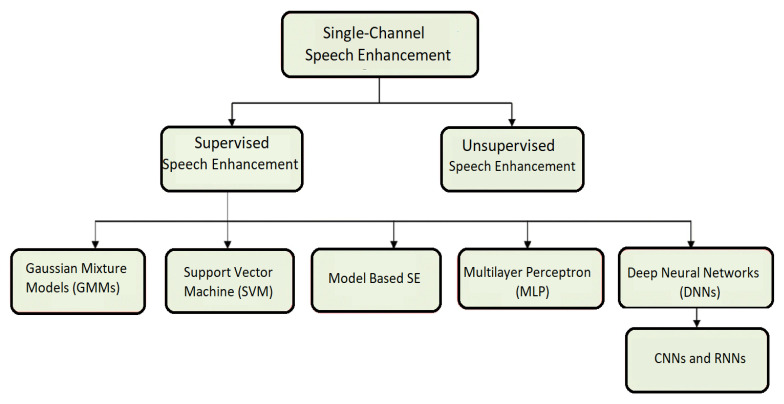
The overall speech enhancement research work highlighting the flow of the work.

The WaveNet model, which was first suggested for use in text-to-speech applications, was also implemented in the waveform-mapping-based SE systems [25,26]. Fully convolutional architectures have the ability to represent the frequency features of speech waveforms more precisely than fully connected architectures because fully convolutional architectures preserve greater local information than fully connected architectures. More recently, it was suggested that a temporal convolutional neural network (TCNN) [10] might properly describe temporal characteristics and carry out SE in the time domain. Some waveform-mapping-based SE techniques [27] employ adversarial loss or perceptual loss to obtain high-level disparities between predictions and their targets. This was conducted in conjunction with the point-to-point loss that was used for optimization. An efficient characterization of sequential and local patterns is a crucial factor to take into account when evaluating the overall performance of the SE algorithms described above that are based on waveform mapping. The high computational cost and model size of RNN may drastically limit its use; despite the fact that the integration of CNN and RNN/LSTM may be a workable solution, RNN’s applicability may be significantly restricted. In this research work, we introduce and extensively explore an E2E waveform mapping-based SE technique that makes use of a unique CRN. This technique improves efficiency by combining the advantages of CNN with parallel recurrent models (LSTM, GRU, and SRU), allowing us to map waveforms from start to finish. In contrast to spectral mapping-based CRN models [14,15], the proposed solutions directly estimate feature masks from unprocessed waveforms using highly parallelizable recurrent networks. A diagram explains the overall speech enhancement research work highlighting the flow of the work is demonstrated in Figure 1.

The remaining portions of the paper are structured as follows: Section 2 presents related studies. The methodology for the proposed E2E waveform-based SE is explained in Section 3. Section 4 presents experiments, whereas results and discussions are given in Section 5. The concluding remarks of this study are drawn from Section 6.

## 2. Related Studies

The majority of existing speech enhancement systems involve spectrogram features [28,29,30], which require a complex transformation and result in phase information loss. Convolutional networks have been used in earlier research to solve these problems by learning the temporal correlation amongst high-resolution speech waveforms. However, the memory-intensive dilated convolution and aliasing problems caused by upsampling restrict the performance of these models. Due to its straightforward design workflow, E2E deep learning models have received a lot of attention for speech enhancement. The local and sequential (speech waveforms) characteristics of speech should be effectively taken into consideration during modeling in order to enhance the performance of an E2E model.

The study [31] presents a completely E2E recurrent neural network (RNN) for enhancing single-channel speech. By lowering the feature resolution without sacrificing the information, an hourglass-shaped network effectively captured long-range temporal correlation. Additionally, the study leveraged residual connections to increase model adaptation and stop gradient deterioration across the layers. According to experimental findings, the E2E-RNN model performs better than cutting-edge techniques in six quantitative performance indicators. The study [21] presents a fully convolutional network (FCN) for waveform-based SE where waveforms have been modeled using convolutional layers. FCN only has convolutional layers, so local temporal speech features are retained with little weights. Experiments reveal that simple DNN and CNN-based models are not able to recover high-frequency waveform components, thereby reducing speech intelligibility. The proposed FCN model recovers waveforms successfully and outperforms the LPS-based DNN baseline in terms of intelligibility and speech quality. The study [32] presents an efficient E2E SE model which employs the CNN module to retrieve speech locality features and the SRU module to represent their sequential properties. SRU can be effectively parallelized in computation, using fewer model parameters than LSTM and GRU. With the SRU and the constrained feature map, the model performs favourably to other latest techniques with decreased computational cost and running time.

A wavenet-based E2E SE is proposed [26], where the suggested model adaption preserves the Wavenet’s outstanding acoustic modeling capabilities while decreasing its temporal complexity. The model uses non-causal, dilated convolutions and predicts target signals. The discriminative model adapts by reducing regression loss with supervised learning. These changes make training and inference parallelizable. Both computational and perceptual assessments recommend the suggested technique above Wiener filtering, which evaluates the magnitude spectrogram. Due to high speech sampling rates, using a lengthy temporal input context at the sample level is challenging yet essential for high-quality SE results. For this, the study [33] presents the Wave-U-Net, which resamples feature maps to calculate and aggregate information at various time scales. With architectural changes, the study provides an additional output layer, an upsampling approach, and a context-aware prediction framework to decrease artifacts. Experiments for speech separation show that the Wave-U-Net architecture performs similarly to a state-of-the-art spectrogram-based U-Net architecture. Finally, the study highlights an issue with outliers in existing SDR assessment criteria and advises presenting rank-based data.

The study [34] presents CNN for real-time SE in the temporal-domain. The suggested CNN uses an encoder-decoder architecture with a temporal convolutional module. The encoder part of temporal CNN low-dimensionalizes a noisy input frame. The temporal convolutional module employs causal and dilated convolutional layers to exploit present and previous frames of encoder output. The Decoder reconstructs improved frames from the outputs. The model is speaker as well as noise-independent and, according to experiments, consistently outperformed the SOTA real-time convolutional recurrent model. Fully convolutional models have fewer trainable parameters than other models. The study [35] proposes the temporal CRN, an E2E neural model that maps the noisy waveforms to the clean waveforms. The model efficiently exploited both short-term and long-term information. In addition, the study offered a forward propagation architecture that downsamples and upsamples the speech waveforms. The proposed model outperformed CRNs and also provided crucial training stabilization approaches. In terms of speech intelligibility and quality, the temporal CRN model exceeded the previous techniques.

The study [36] examined how the loss functions affect the time-domain deep learning SE. Perceptually inspired loss functions may be better than MSE. The study demonstrated that the learning rate is a significant design parameter even for adaptive gradient-based optimizers, which is typically disregarded. In addition, waveform matching performance measurements may fail totally in certain cases. Finally, it has been demonstrated that a loss function based on scale-invariant signal-to-distortion ratio yields strong overall performance across a variety of common SE assessment metrics, suggesting that signal-to-distortion ratio is a solid general-purpose loss function for SE systems. The study [23] presents an E2E utterance-based SE framework employing FCNs. Due to utterance-based optimization, temporal correlation information is used to directly improve the perception-based objective measures. The FCN is utilised to optimise speech intelligibility. Due to consistency between training and assessment measures, the experimental findings have suggested that the proposed SE improves the intelligibility over standard MSE-optimized speech. By adding intelligibility into model optimization, human subjects and automated ASRs can understand the enhanced speech better than with the least MSE criteria.

Using generative adversarial networks (GANs) on the raw signal, the study [27] offers a generative technique to regenerate noisy signals into their clean versions. Different variants of the proposed system are investigated to determine the best architecture for an adversarially trained convolutional auto-encoder applicable for speech signals. The suggested approach is objectively and subjectively evaluated. The former lets us pick among variants and tweak hyperparameters, while the latter is employed in a 42-subject listening experiment to confirm the approach’s success. In addition, showed how the method may be used to regenerate whispered speech. The research [37] offers time-domain SE using GAN, an extension of the generative adversarial network in the time-domain with metric assessment to alleviate the scale issue and give model training stability, thereby improving performance. In addition, provides a novel approach based on objective function mapping to analyse Metric GAN’s performance and explain why it is superior to Wasserstein GAN. Experiments prove that the suggested technique works and show the benefits of Metric GAN. Table 1 summarizes the various neural models with research gap for SE.

In this paper, we propose and thoroughly examine an E2E waveform mapping-based SE approach utilising an alternative CRN. This method achieves better efficiency by combining the benefits of CNN and parallel recurrent models (LSTM, GRU, and SRU), which enables us to map waveforms from end-to-end. In contrast to CRN models that are based on spectral mapping, the proposed methods directly estimate feature masks from unprocessed waveforms using highly parallelizable recurrent networks. The contributions of this study include: (a) Unlike CRNs proposed in [14,15] based on spectral mapping, the proposed E2E-models directly generate feature masks from raw waveforms using highly parallelizable recurrent modules. For SE, we have examined our methodology using accessible datasets [38,39,40] and obtained high speech quality ratings equivalent to the state-of-the-art technique while using a very straightforward architecture and l1 loss function. (b) There is no need for handmade acoustic features or their processing while using raw speech waveforms as model inputs. Furthermore, no linear interpolation techniques are needed for upsampling, which might result in the loss of essential information. The suggested E2E-model is a simple design which outperforms a number of complex neural network techniques. This architecture, we believe, may be used for regression challenges other than speech enhancement, which involves long-term dependency and high-resolution time-series data. We examined our E2E model using various objective measures, confirming its potential to greatly improve the voice quality and intelligibility.

## 3. Proposed E2E Waveform-Based SE Algorithm

**Figure 2:**
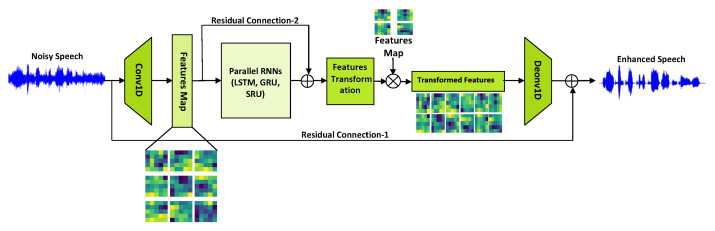
Architecture of the proposed E2E model. It integrates 1D-CNN with bidirectional RNNs: Long Short-Term Memory (LSTM), Gated Recurrent Unit (GRU), Simple Recurrent Unit(SRU). There are two residual connections; (i) adding recurrent net input to recurrent net output and (ii) adding input to the Deconv-1D layer output.

This section describes the proposed E2E SE system in detail. The architecture is a completely discrete E2E neural network without any preprocessing or customized acoustic features. It jointly represents local and sequential information by leveraging the benefits of CNN and parallel RNNs. Figure 2 illustrates the model’s general structure of the proposed SE algorithm.

Our model has adopted the 1D CNN input module for SE implementation based on waveform mapping. WaveCRN [18] is the foundation for these SE models. For feature map extraction, the frames of input noisy speech and two-dimensional (2D) tensors are convolved. The convolution stride is selected to half the kernel size to decrease the length of the feature map. With such arrangements, the feature map is reduced from speech length to time steps in order to properly compute sequences. Following the 1-D convolutional layer (Conv-1D), there is a batch normalization (BN), PReLU activation, Bi-LSTM/Bi-GRU/Bi-SRU modules, and a 1-D deconvolutional layer (Deconv-1D). Conv-1D with Recurrent Net is an effective module for transforming noisy waveforms to clean waveforms. Convolution and recurrent networks may process speech at the frame and utterance levels, respectively. Three types of temporal encoders are used for this purpose: the bidirectional LSTM (Bi-LSTM), the bidirectional GRU (Bi-GRU), and the bidirectional SRU (Bi-SRU). Bi-LSTM, Bi-GRU, and Bi-SRU-based feature extractors are used to construct encoded features for all batches of feature maps. It is applied to the feature maps using a restricted feature mask (RFM). Bi-LSTM/Bi-GRU/Bi-SRU then encodes feature maps into restricted feature masks (RFM), which are element-wisely multiplied by feature maps to generate a masked feature map. There are two residual connections; (i) adding the recurrent net input to the recurrent net output and (ii) adding the input to the Deconv-1D layer output. These residual connections, we discovered, are important for developing a deep neural architecture. Finally, a transposed 1D convolution layer estimates the improved waveform y from the masked feature map.

Usually, the short-time Fourier Transform (STFT) is used to transform speech waveforms into the spectral domain in the case of spectral mapping-based SE systems. However, to perform waveform mapping-based SE, we replaced the STFT processing with a 1D CNN module. Different local patterns of speech signals are captured by a 1D convolutional module. Various feature maps relate to various periodic signal elements. In terms of signal processing, convolutional kernels can be considered as a collection of finite-impulse-response (FIR) filters. Convolutional kernels have the capacity to resemble ordinary filter banks [20]. The outputs of the time-convolution are thus viewed as a concealed Time-Frequency (T-F) representation. The CNN module is completely trainable owing to the nature of neural nets. The input noisy audio $Y\in R^{N\times 1\times L}$ is convolved with a two-dimensional tensor $M\in R^{C\times K}$ for every batch to extract the feature map $M\in R^{N\times C\times T}$ with the batch size *N*, channels number *C*, size of kernel *K*, time steps *T*, and speech length *L*, respectively. Furthermore, in order to limit the sequence length for computational performance, the convolution stride was set to half the size of the kernel size, resulting in reducing the length of M from *L* to $T=\frac{2L}{K+1}.$

**Figure 3:**
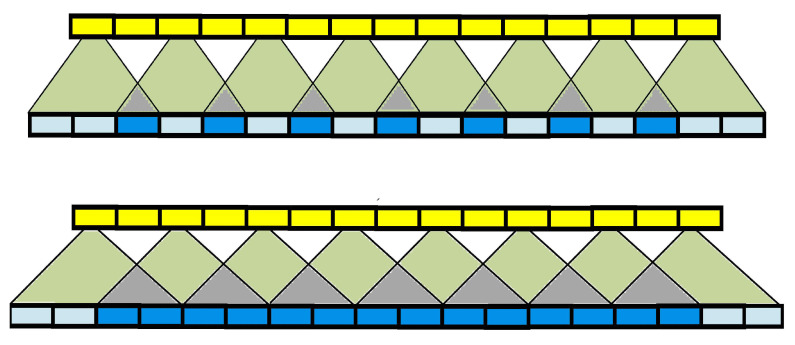
Upper Panel: A 1-D Deconv with uneven overlaps, where kernel size K=3 and stride λ=2. Bottom Panel: A 1-D Deconv with even overlaps, for K=4 and stride λ=2. The light blue units are the results of an upsampling operation, where dark blue units represent overlapped upsampling.

With a high computational load, RNN-based SE models can obtain good results [19]. Therefore, various recurrent models are used in this study to examine SE performance when combined with CNN. We captured and examined the temporal correlation of the feature maps extracted by the input module in both directions using Bi-LSTM, Bi-GRU, and Bi-SRU. The feature maps are passed through the LSTM/GRU/SRU-based recurrent feature extractor for each batch. The encoded features are formed by concatenating the hidden states extracted in both directions. The feature maps are multiplied to the restricted mask $Q\in R^{N\times C\times T}$ to transform the feature maps. With 1D temporal deconvolution (Deconv-1D), we upsampled the features back to raw waveforms. The deconvolutional layer enables the model to construct a waveform segment using the transformed features vector. However, this process is prone to uneven overlaps, resulting in an unusual pattern of distortions, shown in Figure 3. When the kernel size is not divisible by the stride, the deconvolutional layer exhibits uneven overlap. For this reason, the stride was set to be half the kernel size to ensure that the outputs are equally balanced and free of distortions. Since the feature map length was reduced, length restoration is required to generate waveforms that have lengths similar to the input waveforms.

Given the input and output lengths as $L_{in}$, $L_{out}$, whereas stride and padding are as λ and γ, the relationship between input and output lengths is expressed as:

(1) $$L_{out}=\left(L_{in}-1\right)\times \left(\lambda -2\right)\times \gamma +\left(K+1\right)+1$$

**Figure 4:**
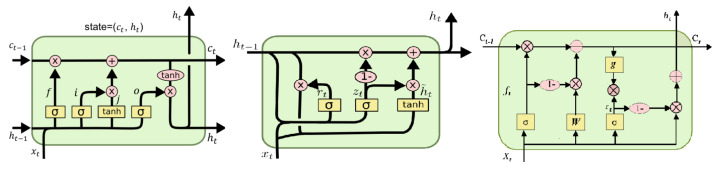
Internal structures of LSTM (**Left**), GRU (**Middle**), and SRU (**Right**).

With $L_{in}=T=\frac{2L}{K+1}$, $\lambda =\frac{K}{2}$, and $\gamma =\frac{K}{2}$, $L_{out}$ is same as *L* which indicates that output waveforms have the same length as input waveforms. The waveform error ${ł}_{we}$ is used as the time-domain loss function. For the output time-domain signal and the corresponding target signal with N samples, the ${ł}_{we}$ is defined as:

(2) $${ł}_{we}=\frac{1}{N}\sum_{j=1}^{N}{\left(x_{i}-{\hat{x}}_{i}\right)}^{2}$$

where ${ł}_{we}$ is the waveform error (loss function), *N* are samples of target speech, $x_{i}$ is input speech and ${\hat{x}}_{i}$ is output (estimated speech). We investigated each of the three RNNs separately and created E2E SE models. To reduce the computational cost of deep models while preserving noise suppression efficacy, RNNs are incorporated to capture temporal correlations. The internal structures of three RNN variations (LSTM, GRU, and SRU) are illustrated in Figure 4. The three E2E SE models are denoted as E2E-BLSTM-CRN, E2E-BGRU-CRN, and E2E-BSRU-CRN, respectively.

The attention mechanism in the residual connections of the proposed model is composed of three components: Query **Q**, Key **K**, and Value **V**. The correlation scores of rows in **Q** are first calculated with all the rows in **K** using the expression, given as:

(3) $$W=QK^{T}$$

where $K^{T}$ is the transpose of **K**. The correlations scores are than converted to the probabilities using the Softmax operator as:

(4) $$Softmax\left(W_{i,j}\right)=\frac{exp^{W_{i,j}}}{\sum_{j=1}^{T-1}exp\left(W_{i,j}\right)}$$

Finally, the rows of V are linearly combined using weights in *Softmax* *(W)* to obtain the attention output.

(5) A=Softmax(W)V

The attention mechanism is termed as self-attention if Q and K are computed from the same sequence.

## 4. Experiments

### 4.1. Datasets

Experiments are carried out to evaluate the performance of the proposed SE by collecting utterances from the TIMIT [39], LibriSpeech [38], and VoiceBank [40] databases, respectively. The clean speech utterances are collected from the databases (TIMIT, LibriSpeech and VoiceBank). With this arrangement, we have created a combined dataset consisting of three separate datasets which has increased the generalization of the dataset. The TIMIT dataset consists of phonetically balanced speech waveforms sampled at 16 kHz, while LibriSpeech has 1000 h of speech waveforms. The Voice Bank database contains 28 speakers from the English accent group (England) and 56 speakers from other English-speaking regions (Scotland and the United States). In our tests, we solely used clean speech utterances from three databases. Noise sources from the Aurora-4 [41], NOISEX-92 [42], and DEMAND [43] databases are used to examine the proposed SE models in noisy environments. Three SNRs (signal-to-noise ratios) ranging from −5 dB to 5 dB with a 5 dB step size are used to generate noisy utterances. SNR is a measure of the strength of the desired speech signal relative to background noise (undesired signal). A collection of utterances is chosen from the TIMIT, LibriSpeech, and VoiceBank databases to train the proposed model. The training utterances comprise both genders and are mixed with all noises to improve speaker generalization. As a result, a large number of utterances from the TIMIT, LibriSpeech, and Voicebank databases are included in model training. The model testing uses a distinct collection of utterances collected at random from the TIMIT, LibriSpeech, and Voicebank databases. All noises, with the exception of two, are used in training and testing. As unseen noises, factory2, and cafe noises are included. Seen noises appear both in training and testing whereas unseen noises are not appeared in the training process.

### 4.2. Evaluation Measures

The experiments use four objective metrics to quantify the suggested SE, including the STOI (short-time objective intelligibility), the PESQ (perceptual evaluation of speech quality), and the composite measures (CM). Quality, Intelligibility, distortion, and residual noise are determined by STOI, PESQ, and CM, respectively. The majority of the objective methods have been proven to be insufficient for evaluating a wide variety of distortions, including those that are often present when speech passes over communication systems.

PESQ [44], an ITU-T P.862 recommendation, scores perceptual speech quality from −0.5 to 4.5. The *PESQ* measure considers positive and negative loudness variations differently, in contrast to other objective measures, which treat both in the same way. This is because the perceived quality is affected differentially by positive and negative loudness variances. A positive difference would suggest the addition of a component to the spectrum, such as noise, while a negative difference would suggest the removal or significant attenuation of a spectral component.The average disturbance value $d_{sym}$ and the average asymmetrical disturbance value $d_{asym}$ are combined linearly to get the final *PESQ* score, as given in Equation (6).

(6) $$PESQ=A_{0}+A_{1}d_{sym}+A_{2}d_{Asym}$$

where $d_{sym}$ and $d_{Asym}$ are symmetric and asymmetric distributions, respectively, whereas $A_{0}$, $A_{1}$, and $A_{2}$ are the parameters with predefined fixed values 4.5, 0.1, and 0.0309, respectively.

STOI [45] assesses speech intelligibility that generates values ranging from 0 to 1. *STOI* presents a correlation between the temporal envelopes of the clean and distorted speech in short-time speech segments. *STOI* is different from many objective measures which usually consider the entire speech signal or use a very short speech segment of 10–20 ms for analysis.

The composite measures [46] is the combination of different measures including $C_{SIG}$ (determines the distortion of speech) and $C_{BAK}$ (determines the residual noise). The reason behind the composite measure is to combine different objective measures to get a strong correlation between signals.

(7) $$C_{SIG}=3.093-1.029LLR+0.603PESQ-0.009WSS$$

(8) $$C_{BAK}=1.634-0.478PESQ-0.007WSS-0.063SSNR$$

where *LLR* is log-likelihood ratio, *WSS* is the weighted spectral slope, and *SSNR* is segmental *SNR*, respectively.

### 4.3. Model Architecture

**Figure 5:**
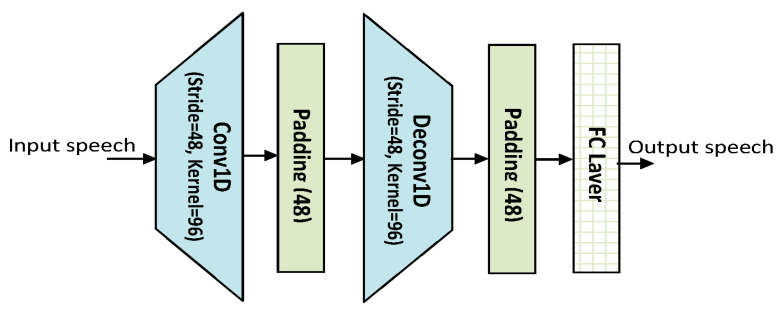
Model Architecture.

The number of channels (*C*), kernel size (*K*), and stride size (λ) in the input Conv-1D module were set to 256, 96, and 48, respectively, with padding (48). Padding was applied to the raw speech signals to make them divisible by the stride size. The number of channels was used to determine the size of the Bi-LSTM/Bi-GRU/Bi-SRU hidden state (6 stacks). To change the masked feature maps, all the hidden states were linearly shifted to a half dimension. Finally, a deconvolutional layer was applied in the waveform generation step to translate the 2D feature maps into a 1D sequence, that was then passed through an activation function to obtain the enhanced speech waveform. The model’s input features are 512 dimensional, whereas the output features are 256 dimensional. Figure 5 demonstrates the model architecture.

## 5. Results and Discussions

This section discusses the results of this study. We examined the proposed E2E SE models objectively, as indicated in the following subsections.

### 5.1. Speech Enhancement in Seen Noises and SNRs

In terms of the *STOI* and *PESQ*, Table 2 compares the proposed SE methods for the four example seen noises. When using the proposed E2E SE models, we noted improved intelligibility and quality compared to the noisy speech. For example, the E2E-BLSTM-CRN increased the *STOI* and *PESQ* over the noisy speech (UNP) at −5 dB babble noise by 23.37% and 36.02%, respectively. Similarly, important improvements in *STOI* and *PESQ* were observed by E2E-BGRU-CRN over the noisy speech at −5 dB exhibition hall noise, thereby improving *STOI* by 27.9% and 35.15%, respectively. E2E-BSRU-CRN improved the *STOI* by 22.24% and 35.51% over noisy speech in the street environment, respectively. The overall *STOI* and *PESQ* in Table 1 for all SNRs and four noises, the E2E-BSRU-CRN, achieved the best scores and improved by 22.62% (*STOI*) and 33.07% (*PESQ*). The other two variants also performed very well in achieving excellent *STOI* and *PESQ*, that is, E2E-BLSTM-CRN achieved 17.35% (*STOI*) and 31.2% (*PESQ*) whereas 21.26% and 31.74% improvements in *STOI* and *PESQ* were obtained with E2E-BGRU-CRN.

**Figure 6:**
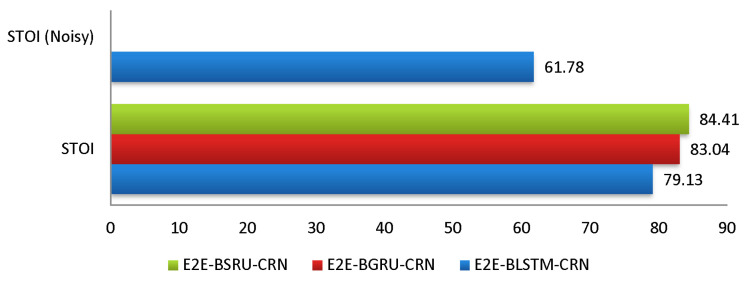
Average *STOI* Scores (Seen Noisy Environments).

**Figure 7:**
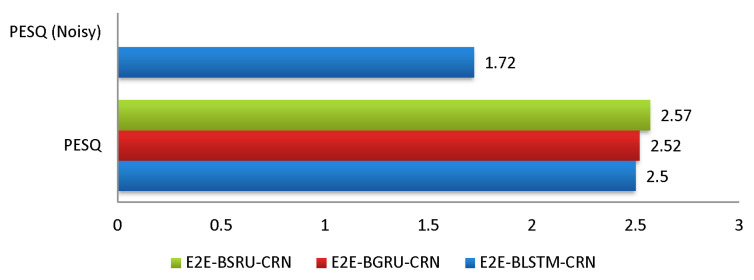
Average *PESQ* Scores (Seen Noisy Environments).

Table 3 summarizes the average *STOI* and *PESQ* scores, averaging the findings across all types of seen noises. The results show unequivocally that E2E-BSRU-CRN accomplished significant outcomes in terms of the *STOI* and PESQ. Figure 3 shows the average improvements (STOIi and PESQi) in different seen noise categories. The average *STOI* and *PESQ* scores in Table 2 show that E2E-BSRU-CRN effectively reduced the noise signals with better speech intelligibility and perceptual quality as compared to its counter E2E models (E2E-BLSTM-CRN and E2E-BGRU-CRN) for speech enhancement. Figure 6 and Figure 7 demonstrates the average *STOI* and *PESQ* scores in seen noisy environments.

Table 4 shows test findings for speech distortion (CSIG) and residual noise distortion (CBAK). It is clear that in terms of residual noise and speech distortion, the proposed CNN and recurrent networks with residual connections outperformed. The background additive noise frequencies were successfully decreased and less speech distortion was caused by all three CRN models (E2E-BLSTM-CR, E2E-BGRU-CRN, and E2E-BSRU-CRN). The E2E-BSRU-CRN, E2E-BLSTM-CRN, and E2E-BGRU-CRN improved the average CSIG and CBAK scores from 1.78 and 1.59 with noisy speech at −5 dB to 2.89, 2.82, and 2.85. This increased the CBAK by factors of 1.12 (38.40%), 1.04 (36.87%), and 1.07 (37.54%), respectively. The CSIG was raised by factors of 0.69 (30.39%), 0.63 (28.50%), and 0.74 (31.89%), respectively, by bringing the average CBAK to 2.27, 2.21, and 2.32. As shown in Table 3, the suggested approaches greatly decreased the residual noise and speech distortion for SNRs other than-5dB. A better CSIG and CBAK scores are obtained with E2E-BSRU-CRN as compared to other two E2E models. The average CSIG and CBAK scores with E2E-BSRU-CRN are improved from 2.02 and 1.73 to 3.10 (34.83%) and 2.66 (34.96%), respectively.

### 5.2. Speech Enhancement in Unseen Noises

Table 5 compares the proposed SE methods for the two example unseen noises. The two noise types (factory2 and cafeteria) were not included in the training. When using the proposed E2E SE models, we noted improved intelligibility and quality compared to the noisy speech. For example, the E2E-BLSTM-CRN increased the *STOI* and *PESQ* over the noisy speech (UNP) at 0 dB factory2 noise by 24.14% and 33.87%, respectively. Similarly, important improvements in *STOI* and *PESQ* were observed by E2E-BGRU-CRN over the noisy speech at 0 dB cafeteria noise, thereby improving *STOI* by 26.44% and 34.84%, respectively. E2E-BSRU-CRN improved the *STOI* by 24.95% and 35.68% over noisy speech in the cafeteria environment, respectively. The overall *STOI* and *PESQ* in Table 4 for all SNRs and two unseen noises, the E2E-BSRU-CRN, achieved the best scores and improved by 24.45% (*STOI*) and 35.11% (*PESQ*). The other two variants also performed very well in achieving excellent *STOI* and *PESQ*, that is, E2E-BLSTM-CRN achieved 16.21% (*STOI*) and 29.34% (*PESQ*), whereas 20.01% and 29.55% improvements in *STOI* and *PESQ* were obtained with E2E-BGRU-CRN.

### 5.3. Comparison with Competing SE Models

**Figure 8:**
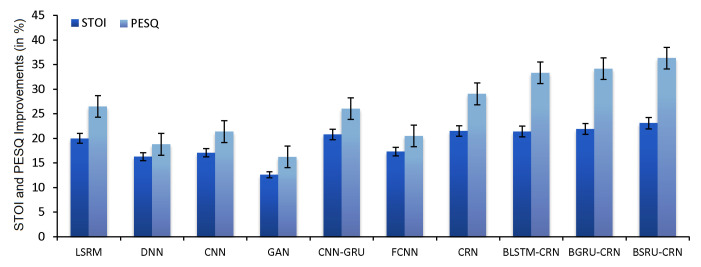
*PESQ* and *STOI* Percentage Improvements of All Speech Enhancement Models over the Noisy speech (Unprocessed).

In this part, we provide the average test results in terms of the *STOI* and *PESQ* for the proposed models and the alternative SE models. The findings demonstrate that the suggested E2E models outperformed the LSTM [47], DNN [48], CNN [49], GAN (3-layer ReLU MLP) [50], CNN-GRU [51], FCNN [52], and CRN [32] models in terms of speech quality and intelligibility. Table 6 provides the generalizations of the proposed and competing models, all of which were trained using the same training and testing data from both gender. For this section of experiments, separate set speech utterances is used to obtain the generalization of the proposed models. The noise types and SNRs are averaged to provide the findings. The results clearly show that the suggested E2E models for SE raised the quality and understandability. For instance, the E2E-BSRU-CRN and E2E-BGRU-CRN increased the average *STOI* over LSTM by 3.1% and 1.9%, respectively. Similarly, the E2E-BSRU-CRN and E2E-BGRU-CRN increased the average *STOI* over DNN by 6.8% and 5.6%, respectively. Additionally, E2E-BSRU-CRN outperformed the CNN and GAN in terms of *STOI* by 10.5% and 6.0%, respectively. When it comes to the *PESQ*, the E2E-BSRU-CRN outperformed the FCNN, GAN and CNN by factors of 0.37 (14.39%), 0.47 (18.28%), and 0.35 (13.61%), respectively. Furthermore, the E2E-BGRU-CRN outperformed the FCNN, DNN and LSTM by factors of 0.32 (12.69%), 0.36 (14.28%), and 0.18 (7.14%), respectively. Figure 8 displays the total average improvement of the proposed and competing models over the noisy speech.

In order to highlight the advantages of supervised learning over unsupervised deep learning, we also compared the proposed models with three unsupervised approaches. Low-rank sparse decomposition (LRSD) [53], Nonnegative RPCA (NRPCA) [54], and MMSE [55] are among some of the unsupervised algorithms for SE Both the LRSD and the NRPCA estimated binary masks. Table 7 displays the test results in terms of *STOI* and *PESQ* for the SE models where the average *STOI* of three E2E-CRN models is raised by 11.2%, 10.9%, and 13.1% over LRSD, NRPCA, and MMSE, respectively. In addition, the *PESQ* results are boosted by factors of 0.49 (19.36%), 0.48 (18.97%), and 0.71 (28.06%), respectively, over unsupervised SE algorithms.

**Figure 9:**
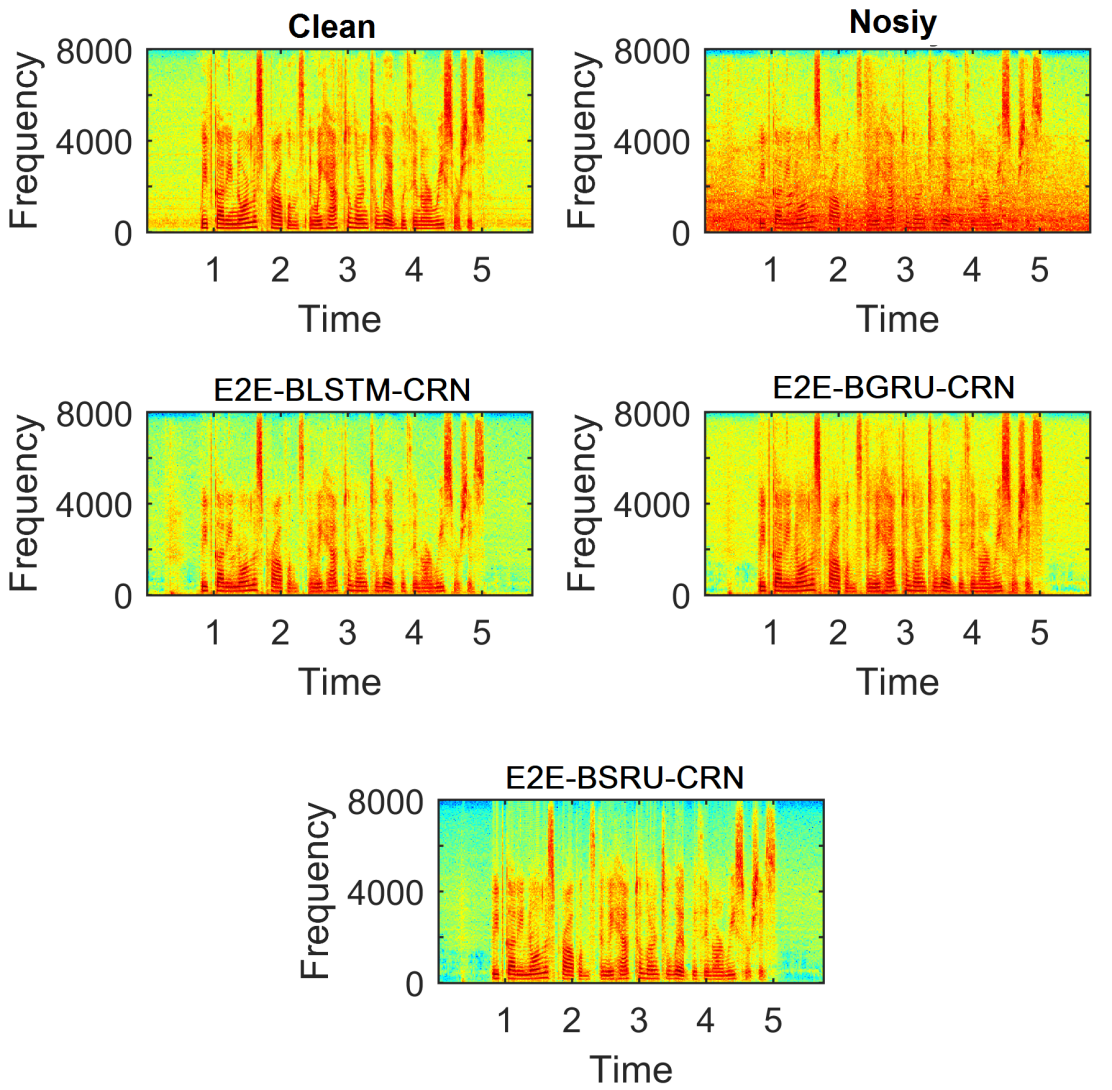
Example Spectrograms of Speech utterance degraded by −5 dB Babble Noise. The spectrograms of three E2E models show less residual noise.

Less distortion and residual noise are evident in the enhanced speech generated by the proposed E2E SE models, as shown in Figure 9. The spectrogram of speech generated using the proposed models shown to have substantially less residual noise and speech distortion. Figure 9 shows example spectrograms of speech signals corrupted by babble noise at −5 dB SNR. There is less significant residual noise in the spectrogram of speech processed by E2E-BSRU-CRN. The other two variants (E2E-BLSTM-CRN and E2E-BGRU-CRN) also reduced the background noise with less speech distortion.

### 5.4. Model Depth

As examined in the above discussions, the performance of the SRU-based RNN is better than that of LSTM and GRU in terms of *STOI* and *PESQ*. To examine the depth of models, we evaluated the training time and trainable parameters of three RNNs. In experiments, we have used 6stacked LSTM/GRU/SRU. The depth of the models has a great impact on the SE performance. As a result, in this experiment we examined the impact of model depth on the training time (computational load). The total number of trainable parameters (in K), forward and back propagation (in ms) for three E2E models and WaveNet [26] are given in Table 8, where E2E-BSRU-CRN indicates the lower number of trainable-parameters and better forward and back propagation results. On the other hand, LSTM contains a large number of trainable parameters. GRU shares 25% less trainable-parameters as compared to LSTM. In the forward/back propagation pass during the training stage, E2E-BSRU-CRN outperforms Wave-U-Net, E2E-BLSTM-CRN, and E2E-BGRU-CRN while using fewer parameters than Wave-U-Net and the other two E2E-CRNs. Table 8 provides the model size and computational cost.

SRU has been shown [17] to provide performance similar to that of LSTM with greater parallelism. Gate dependencies in LSTMs enable training and inference to be slower. The sequential correlation is represented by adding highway connection across the recurrent layers, while all the gates in SRU solely rely on the input of the present time. As a result, the SRU gates are determined sequentially. SRU and LSTM have forward propagation and time complexity as O(T N C) and O(T N C2), respectively. Due to the benefits listed above, SRU is a good candidate for CNN integration.

### 5.5. Automatic Speech Recognition

**Figure 10:**
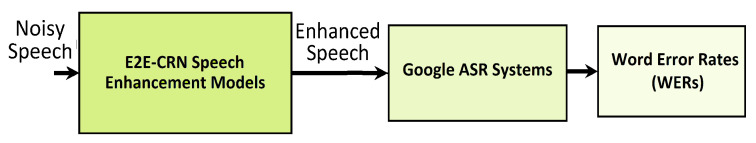
Google ASR system with Speech Enhancement at Front-End.

According to the speech enhancement assessments, the suggested E2E-CRN models significantly reduced the background noise and restored a high-quality, recognizable speech. Therefore, we expected improved speech recognition performance in adverse noise settings. As shown in Figure 10, the suggested SE models are applied at the front-end to provide superior ASR results. We used the Google ASR [56] for this task.

**Figure 11:**
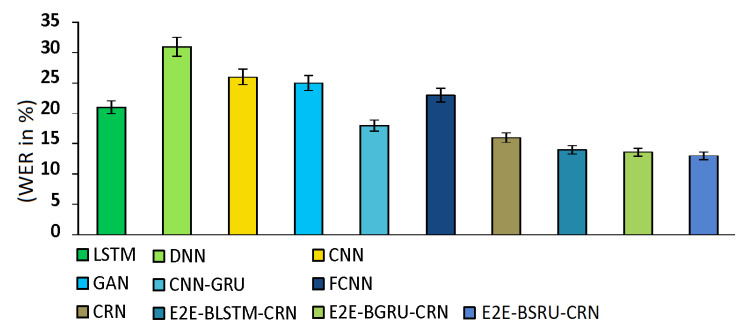
WERs using Google ASR system.

We assessed ASR performance using word error rates (WERs). To train the suggested E2E-CRN speech improvement models, 2000 speech utterances were randomly chosen from the TIMIT and LibriSpeech datasets. We enhanced speech using the trained models, and then we created new training and testing datasets by generating time-domain utterances. Both the new training dataset and the new testing dataset were used to test the ASR models. As shown in Figure 11, the ASR system outperformed other SE models when evaluated using utterances processed by E2E-CRN models. With the good SNR levels, the WERs steadily dropped. The suggested SE may be used as a front-end to improve the ASR performance as seen by the average 13% WERs obtained with the utterances processed by the proposed E2E-CRNs.

### 5.6. Cross Corpus Analysis

To investigate how well the proposed neural models generalise across the corpora, we conducted an experimental investigation. The speech quality and clarity of three datasets—TIMIT, LibriSpeech, and VoiceBank—are investigated. A speech dataset is often made up of multiple utterances made by different speakers. The spoken utterances are recorded in restricted settings for clear recordings that are suitable for speech applications. Speech utterances may have distinct components as a result of the utterances being captured in preference contexts for various datasets. For instance, the quality of an utterance collected by the same individual using several microphones might vary greatly. We give Table 9 which presents the average *PESQ* and *STOI* values across all noise types and SNR levels to investigate the impact of the deep neural models for various speech datasets. The same number of training utterances are used for all models, and they are all subsequently assessed using the same collection of utterances. The cross-corpus findings show that suggested and other deep models outperformed LibriSpeech and TIMIT when trained with the VoiceBank dataset. The average of three E2E models is represented as E2E-CRNs for simplicity.

## 6. Conclusions

For improving degraded speech, end-to-end deep learning models have attracted a lot of interest. The local and sequential attributes of speech signal should be effectively taken into consideration while modelling in order to enhance the performance of E2E models. We have developed resource-effective and compact neural models for waveform-based end-to-end speech enhancement that are noise-resistant. We developed three distinct speech enhancement systems based on LSTM, GRU, and SRU by fusing the Convolutional Encode-Decoder (CED) and Recurrent Neural Networks (RNNs) in the Convolutional Recurrent Network (CRN) architecture.the experiments show that the proposed models lead to improved quality and intelligibility with fewer trainable parameters, notably reduced model complexity, and inference time than existing recurrent and convolutional models. The E2E-BLSTM-CRN increased the *STOI* and *PESQ* over the babble noisy speech by 23.37% and 36.02%, respectively. Important improvements in *STOI* and *PESQ* were observed by E2E-BGRU-CRN over the noisy speech in exhibition hall noise, thereby improving *STOI* by 27.9% and 35.15%, respectively. The findings also concluded that the suggested E2E models outperformed the LSTM, DNN, CNN, FCNN, CNN-GRU and GAN models in terms of speech intelligibility and quality. Less distortion and residual noise are concluded in the enhanced speech generated by the proposed E2E SE models. It is also concluded that the ASR system outperformed other SE models when evaluated using utterances processed by E2E-CRN models. In the forward/back propagation pass during the training stage, E2E-BSRU-CRN outperforms Wave-U-Net, E2E-BLSTM-CRN, and E2E-BGRU-CRN while using less parameters than Wave-U-Net and the other two E2E-CRNs. Finally, the cross-corpus findings show that suggested and other deep models outperformed LibriSpeech and TIMIT when trained with the VoiceBank dataset.

Phase is an important aspect of modern speech enhancement systems since phase plays a significant role in improving the speech quality. This paper emphasize the speech magnitude enhancement. We will be devoted to include the phase estimation [57] and incorporate with the proposed SE model. Moreover, more robust loss functions will be worked out for better results.

## Figures and Tables

**Table 1:** Summary of Related Studies.

Reference#	Neural Model	Processing Domain	Research Gaps
[28]	CNN + LSTM	Frequency-Domain	Handy-crafted features with no phase estimation. High computational Load
[29]	DNN + EMD	Frequency-Domain	Handy-crafted features with no phase estimation, High computational Load
[30]	DNN, RNN	Frequency-Domain	Handy-crafted features with iterative phase estimation but high computational Load
[31]	GRU, RNN	Time-Domain	No handy-crafted features, requires no phase estimation, Computationally efficient
[32]	CNN + BiSRU	Time-Domain	No handy-crafted features, requires no phase estimation, Computationally efficient
[33]	CNN	Time-Domain	No handy-crafted features, computationally efficient but no spectral analysis is performed
[34]	CNN	Time-Domain	No handy-crafted features, computationally efficient but encoder-decoder architecture with various skip connections
[35]	CNN	Time-Domain	No handy-crafted features, requires no phase estimation, but computationally not efficient
[37]	GAN	Time-Domain	Generative networks which require high computational load

**Table 2:** *STOI* and *PESQ* scores in example Seen Noises.

Noise Type	Model	*STOI*			*PESQ*		
		−5 dB	0 dB	5 dB	−5 dB	0 dB	5 dB
Babble Noise	Noisy (UNP)	48.20	58.10	67.11	1.35	1.71	2.04
	E2E-BLSTM-CRN	71.57	82.88	90.11	2.11	2.55	2.80
	E2E-BGRU-CRN	73.06	81.76	91.23	2.09	2.58	2.83
	E2E-BSRU-CRN	74.93	83.09	90.59	2.13	2.61	2.89
Exhibition Noise	Noisy (UNP)	51.09	58.91	68.61	1.42	1.66	1.96
	E2E-BLSTM-CRN	77.96	79.59	78.58	2.19	2.57	2.82
	E2E-BGRU-CRN	79.71	85.41	87.77	2.22	2.58	2.84
	E2E-BSRU-CRN	79.66	86.88	88.24	2.23	2.62	2.90
Street Noise	Noisy (UNP)	55.21	61.70	69.79	1.39	1.77	2.16
	E2E-BLSTM-CRN	75.77	83.81	91.19	2.09	2.56	2.79
	E2E-BGRU-CRN	75.85	84.12	91.01	2.13	2.57	2.84
	E2E-BSRU-CRN	77.44	84.76	91.31	2.15	2.60	2.87
Restaurant Noise	Noisy (UNP)	54.96	66.54	68.52	1.41	1.73	2.09
	E2E-BLSTM-CRN	75.37	83.72	89.59	2.13	2.58	2.83
	E2E-BGRU-CRN	75.03	83.74	87.73	2.15	2.61	2.84
	E2E-BSRU-CRN	78.24	87.04	91.13	2.21	2.65	2.92

**Table 3:** Average *STOI* and *PESQ* scores in all Seen Noises.

Measure	Model	−5 dB	0 dB	5 dB	Average
*STOI*	Noisy (UNP)	55.51	61.31	68.52	61.78
	E2E-BLSTM-CRN	70.17	79.85	87.37	79.13
	E2E-BGRU-CRN	75.92	83.76	89.43	83.04
	E2E-BSRU-CRN	77.56	85.34	90.31	84.41
*PESQ*	Noisy (UNP)	1.39	1.72	2.06	1.72
	E2E-BLSTM-CRN	2.13	2.57	2.81	2.50
	E2E-BGRU-CRN	2.14	2.59	2.84	2.52
	E2E-BSRU-CRN	2.18	2.62	2.90	2.57

**Table 4:** Average CSIG and CBAK scores in all Seen Noises.

Measure	Model	−5 dB	0 dB	5 dB	Average
CSIG	Noisy (UNP)	1.78	2.22	2.69	2.23
	E2E-BLSTM-CRN	2.85	3.22	3.74	3.27
	E2E-BGRU-CRN	2.82	3.19	3.71	3.24
	E2E-BSRU-CRN	2.89	3.31	3.81	3.34
CBAK	Noisy (UNP)	1.59	1.83	2.14	1.85
	E2E-BLSTM-CRN	2.27	2.65	2.92	2.61
	E2E-BGRU-CRN	2.21	2.60	2.86	2.56
	E2E-BSRU-CRN	2.32	2.68	2.98	2.66

**Table 5:** *STOI* and *PESQ* scores in Unseen Noises.

Noise Type	Model	*STOI*			*PESQ*		
		−5 dB	0 dB	5 dB	−5 dB	0 dB	5 dB
Factory2 Noise	Noisy (UNP)	47.68	57.80	66.96	1.31	1.64	1.99
	E2E-BLSTM-CRN	71.04	81.94	89.92	2.02	2.48	2.74
	E2E-BGRU-CRN	72.53	81.42	91.05	2.00	2.51	2.77
	E2E-BSRU-CRN	74.40	82.75	90.39	2.05	2.55	2.82
Cafeteria Noise	Noisy (UNP)	51.32	58.61	68.45	1.33	1.51	1.91
	E2E-BLSTM-CRN	77.43	79.25	78.40	2.11	2.48	2.76
	E2E-BGRU-CRN	79.18	85.05	87.58	2.13	2.58	2.78
	E2E-BSRU-CRN	79.11	86.15	88.06	2.16	2.56	2.86

**Table 6:** Comparison with other SE Models.

SE Models	*STOI*				*PESQ*			
	−5 dB	0 dB	5 dB	Avg	−5 dB	0 dB	5 dB	Avg
Noisy (UNP)	55.5	61.3	68.5	61.8	1.39	1.72	2.06	1.72
LSTM [47]	74.2	82.4	88.9	81.8	2.03	2.33	2.67	2.34
DNN [48]	70.0	78.7	85.6	78.1	1.75	2.19	2.53	2.16
CNN [49]	70.0	79.8	86.8	78.9	1.83	2.25	2.59	2.22
GAN [50]	65.0	75.7	82.6	74.4	1.72	2.15	2.44	2.10
CNN-GRU [51]	74.6	83.1	90.1	82.6	2.01	2.34	2.65	2.33
FCNN [52]	71.6	79.3	86.3	79.1	1.78	2.21	2.59	2.20
CRN [32]	76.4	84.2	89.3	83.3	2.04	2.40	2.73	2.40
E2E-BLSTM-CRN	75.2	83.8	90.4	83.2	2.13	2.57	2.81	2.50
E2E-BGRU-CRN	75.9	84.3	90.9	83.7	2.14	2.59	2.84	2.52
E2E-BSRU-CRN	77.6	85.5	91.6	84.9	2.18	2.62	2.90	2.57

**Table 7:** Comparison against Unsupervised Deep Learning.

Measure	SE Algorithms	−5 dB	0 dB	5 dB	Average
*STOI*	Noisy (UNP)	55.51	61.31	68.52	61.78
	LRSD [53]	63.2	70.6	79.43	71.0
	NRPCA [54]	63.3	70.4	80.3	71.3
	MMSE [55]	60.5	68.8	78.1	69.1
	E2E-CRN (Proposed)	74.5	82.9	89.0	82.2
*PESQ*	Noisy (UNP)	1.39	1.72	2.06	1.72
	LRSD [53]	1.71	1.98	2.28	2.04
	NRPCA [54]	1.78	2.02	2.33	2.05
	MMSE [55]	1.51	1.88	2.15	1.82
	E2E-CRN (Proposed)	2.15	2.59	2.85	2.53

**Table 8:** Model Depth and computational cost.

Model	E2E-BLSTM-CRN	E2E-BGRU-CRN	E2E-BSRU-CRN	Wave-UNet
Forward Pass	37.5 ± 2.0	28.12 ± 1.22	2.15 ± 0.006	23.77 ± 0.009
Back Pass	58.47 ± 1.05	43.48 ± 0.22	4.98 ± 0.005	23.76 ± 0.20
Parameters	9203	6902	4976	17537

**Table 9:** Cross Corpus Analysis of *STOI* and *PESQ* scores.

Models	LibriSpeech		TIMIT		VoiceBank	
	*STOI*	*PESQ*	*STOI*	*PESQ*	*STOI*	*PESQ*
E2E-CRNs	79.7	2.29	79.2	2.31	81.1	2.39
LSTM	78.4	2.17	77.3	2.18	78.5	2.27
DNN	71.9	2.05	70.8	1.94	72.0	2.09
CNN	75.1	2.18	74.0	2.07	75.2	2.22
GAN	71.1	2.07	70.0	1.96	71.2	2.11
CNN-GRU	79.0	2.30	77.9	2.19	79.1	2.34
FCNN	75.4	2.16	74.3	2.05	75.5	2.20
CRN	78.1	2.25	77.5	2.20	78.7	2.29

## Data Availability

Not applicable.
